# Cocaine Use Disorder Is Associated With Changes in Th1/Th2/Th17 Cytokines and Lymphocytes Subsets

**DOI:** 10.3389/fimmu.2019.02435

**Published:** 2019-10-15

**Authors:** Aline Zaparte, Jaqueline B. Schuch, Thiago W. Viola, Talita A. S. Baptista, Amanda Stephanie Beidacki, Carine H. do Prado, Breno Sanvicente-Vieira, Moisés E. Bauer, Rodrigo Grassi-Oliveira

**Affiliations:** ^1^Developmental Cognitive Neuroscience Lab, School of Medicine, Pontifícia Universidade Católica do Rio Grande do Sul, Porto Alegre, Brazil; ^2^Graduate Program in Biomedical Gerontology, School of Medicine, Pontifícia Universidade Católica do Rio Grande do Sul, Porto Alegre, Brazil; ^3^Developmental Cognitive Neuroscience Lab, School of Health Science, Pontifícia Universidade Católica do Rio Grande do Sul, Porto Alegre, Brazil; ^4^Laboratory of Stress Immunology, School of Sciences, Pontifícia Universidade Católica do Rio Grande do Sul, Porto Alegre, Brazil

**Keywords:** addiction, cocaine, crack, cytokines, lymphocyte subsets, craving

## Abstract

**Background:** Cocaine is a psychostimulant drug with high addictive proprieties. Evidence suggests that cocaine use leads to critical changes in the immune system, with significant effects on T, B, and natural killer (NK) cells and influencing peripheral levels of cytokines. The presence of abstinence-related symptoms during detoxification treatment is known to influence the prognosis. Here, our aim was to investigate immune profiles in women with cocaine use disorder (CUD) according to withdrawal symptoms severity.

**Methods:** Blood samples and clinical data were collected at onset of detoxification treatment of 50 women with CUD. The patients were stratified according to Cocaine Selective Severity Assessment (CSSA) scores in low withdrawal (L-W) and high withdrawal (H-W) categories. In addition, we also included a control group with 19 healthy women as reference to immune parameters. Peripheral blood was collected and lymphocyte subsets were phenotyped by multi-color flow cytometry (B cells, CD4^+^ T, CD8^+^ T, NK cells, and different stages of T-cell differentiation). PBMCs from patients and healthy controls were stimulated *in vitro* with phytohemagglutinin (1%) for 72 h to assess the production of Th1/Th2/Th17 cytokines.

**Results:** Following stimulation, lymphocytes from women with CUD produced increased levels of Th1/Th2/Th17 cytokines. However, higher levels of IL-2 and IL-17 were observed only in the L-W group, while higher levels of IL-6 were detected in the H-W group compared to controls. H-W group showed lower percentage of early-differentiated Th cells (CD4^+^CD27^+^CD28^+^), elevated percentage of Th cells (CD3^+^CD4^+^), intermediate-differentiated Th cells (CD4^+^CD27^−^CD28^+^), and B cells (CD3^−^CD19^+^). Both CUD groups showed decreased percentages of naïve T cells (CD3^+^CD4^+^CD45RA^+^ and CD3^+^CD8^+^CD45RA^+^).

**Conclusion:** Our data demonstrated that CUD can lead to increased production of Th1/Th2/Th17 cytokines and lymphocyte changes.

## Introduction

Drug dependence is a serious problem for public health worldwide, with higher use estimates in the Americas (1). Among the psychostimulant drugs, cocaine use disorder (CUD) is associated with greater physiological alterations, given that it acts blocking dopamine, serotonin and norepinephrine reuptake (2, 3). Furthermore, cocaine triggers cellular toxicity through different mechanisms, including immune dysregulation (4, 5) and oxidative damage (6, 7), potentially leading to neurological, emotional, and cognitive impairments (8, 9). Studies also observed higher risk of contracting or transmitting infectious diseases among cocaine users compared to non-users (10–12).

The remarkable involvement of the immune system in CUD is also evidenced by the interaction of cocaine with dopamine receptors expressed in immune cells, such as T and B lymphocytes and NK cells (13). These receptors are able to regulate different cellular processes, including apoptosis, proliferation, and differentiation (14). In addition, an immunomodulatory effect of cocaine has been reported, characterized by an increase in NK cells, thereby relatively reducing T CD4^+^ cells (15).

Moreover, an imbalance in the inflammatory profile was described in CUD. In this sense, increased plasma pro-inflammatory molecules, mainly IL-6 and TNF-α, and decreased anti-inflammatory molecules were described in cocaine users when compared to healthy controls (16, 17). Also, results of a previous study conducted by Irwin et al. showed that monocytes obtained from cocaine users during early abstinence presented lower expression of IL-10 and IL-6 at the baseline and also after *in vitro* stimulation with LPS than control subjects, suggesting a significant decrease in innate immune response in cocaine users (18). Lewandowski et al. (19), assessed plasma Th1/Th2/Th17-related cytokines in women with CUD during a 3-week detoxification program. Lower plasma levels of Th1 and Th17 cytokines in the first week of detoxification were observed in women with CUD when compared to the control group. This profile, however, changes at the end of treatment. Interestingly enough, it was found that women with CUD and history of childhood maltreatment (CM) displayed a profound increase in TNF-α levels. IL-6 was also elevated in women with history of CM and diminished in women without CM in comparison with the control group. Now looking at the Th2 profile, IL-4 and IL-10 showed higher expression in smoked cocaine users than in controls (19).

Araos et al. (20) identified a correlation between alterations in proinflammatory components and cocaine abuse. They suggested that cocaine users can be stratified according to cocaine symptom severity and presence of psychiatric comorbidities. This strategy allows proinflammatory markers to be used as predictors of cocaine dependence. Another study showed that, during detoxification treatment, patients may present behaviors attributed to the abstinence period, including anxiety, irritability, depression, attention, and memory deficits, as well as respiratory and circulatory impairments (21–23). It is already known that some of these behaviors are associated with neuroinflammation in different neurodegenerative diseases (24) and psychiatric disorders (25, 26). In the case of substance abuse, the appropriate assessment of such behaviors seems to be extremely critical for predicted success or failure with regard to treatment.

Here, we assessed abstinence symptoms using the Cocaine Selective Severity Assessment (CSSA) to categorize CUD patients with high or low-withdrawal severity. Previous studies showed that individuals with higher CSSA scores exhibit severe addictive behavior and are more likely to discontinue treatment (27). In order to explore the immune alterations caused by smoked cocaine use, peripheral blood mononuclear cells (PBMCs) were stimulated *in vitro* to evaluate the production of Th1, Th2, and Th17 cytokines. Also, a comprehensive panel of lymphocyte subsets (B cells, CD4^+^ T, CD8^+^ T, NK cells, and different stages of T-cell differentiation) was analyzed by multi-color flow cytometry.

The present study focuses on female CUD patients based on clinical studies showing that women: (1) start using cocaine earlier than men (28); (2) report higher amounts of cocaine consumption when compared to men seeking treatment (29); (3) report higher craving and withdrawal symptoms to cocaine than men, and are more vulnerable to develop CUD, showing greater drug use escalation (1).

## Materials and Methods

### Subjects

The CUD samples were recruited at an inpatient unit from Southern Brazil and comprises 50 female adults facing detoxification treatment. Patients included in this study were 18 years or older, had positive urine test for cocaine and fulfilled the diagnosis of CUD. Exclusion criteria includes pregnancy and diagnoses of any neurologic, infectious, inflammatory, or metabolic diseases. Diagnosis of CUD was carried out by trained psychiatrists and psychologists through semi-structured clinical interview, the Structured Clinical Interview for DSM-5 (SCID-5) (30).

Non-addicted individuals were recruited through advertisement in social media and all were screened for drug abuse or dependence. This sample includes 19 female adults (>18 years old), with negative urine test for illicit drugs (cocaine, THC, amphetamine, and opioids) and no history of crack, cocaine, or any other substance use, except for alcohol and nicotine. The exclusion criteria used in the CUD sample was also applied in the control sample. In addition, we chose to include only controls with low or medium socioeconomic status (SES) to match the SES background of the clinical group. Controls were free of anti-inflammatory medications for at least 30 days at time of blood collection. This project was carried out in accordance with the Declaration of Helsinki and all subjects signed an informed consent form previously approved by the Research Ethics Committees of the participating institutions.

### Clinical Assessment

During the first week of the detoxification treatment, a clinical protocol that included the Addiction Severity Index 6 (ASI-6) (31), the Structured Clinical Interview for DSM-5 (SCID-5) (30), and the Cocaine Selective Severity Assessment (CSSA) (27) was performed in the CUD sample. The ASI-6 is a semi-structured interview and it was used to assess the patterns of substance use behavior (crack-cocaine, tobacco, alcohol, and cannabis), including data regarding the age of experimentation of licit and illicit drugs, as well as data on recent substance consumption (last 30 days before treatment enrollment). The ASI-6 was also performed in non-addicted controls.

The CSSA was used to assess the severity of crack-cocaine withdrawal symptoms. Each of the 18 individual items of the CSSA is scored on a 0–7 scale, in which 0 represents no symptoms and 7 represents maximum severity. The signs and symptoms included on the CSSA assessment were crack-cocaine craving, appetite changes, sleep disturbances, lethargy, depressed mood, and bradycardia, which are manifestations that commonly occur after abrupt cessation of crack-cocaine use. The CSSA total score was computed by the sum of each individual item score.

### Isolation of Peripheral Blood Mononuclear Cells (PBMCs)

Peripheral blood (10 mL) was collected from each participant 4 days after the beginning of the detoxification treatment by venipuncture in EDTA tubes. Peripheral blood mononuclear cells (PBMCs) were isolate by Ficoll density gradient centrifugation (Ge Healthcare Life Sciences—Marlborough, MA, USA), 30 min at 900 g. Cells were counted using a microscope (100x) and viability always exceeded 95%, as judged by Trypan Blue exclusion (Sigma-Aldrich—St. Louis, MO, USA).

### Analysis of Secreted Cytokines (Th1/Th2/Th17) *in vitro*

To determine cytokine production, the PBMCs were seeded (1 × 10^5^ cells) in 96-well plates containing RPMI media supplemented with 10% of fetal bovine serum. Cells were stimulated with phytohemagglutinin (PHA) at 1% concentration for 72 h, at 37°C in 5% CO_2_. PHA is a polyclonal mitogen widely used in immunological studies, triggering the T-cell activation, cytokine production and proliferation *in vitro* (32). Supernatants were collected and multiple cytokines levels (IL-2, IL-10, IL-4, IL-6, IFN-γ, TNF-α, and IL-17) were simultaneously determined by flow cytometry (FACS Canto II, BD Biosciences, San Jose, CA, USA) using the Cytometric Bead Arrays (CBAs, Human Th1/Th2/Th17 Kit, BD Biosciences). The instrument has been checked for sensitivity and overall performance with Cytometer Setup & Tracking beads (BD Biosciences) prior to data acquisition. Results were analyzed using the FCAP Array v1.0.1 software (Soft Flow—Pecs, Hungary). The detection limits for these assays ranged from 2.4 to 4.9 pg/ml (IL-2, IL-10, IL-4, IL-6, IFN-γ, TNF-α) and 18.9 pg/ml for IL-17. The intra-assay and inter-assay coefficients of variation were <10%.

### Immunophenotyping

A comprehensive panel of lymphocyte subsets was identified by multicolor flow cytometry. PBMCs were washed in flow cytometry buffer (PBS containing 1% FCS and 0.01% sodium azide) and treated with Fc Block solution for 20 min. Cells were stained for 30 min at 4°C with combinations of monoclonal antibodies: anti-CD3, anti-CD4, anti-CD8, anti-CD45RA, anti-CD45RO, anti-CD19, anti-CD25, anti-CD27, anti-CD28, anti-CD56, and anti-CD69. The fluorochromes used were FITC, APC, and PE and all antibodies were purchased from BD Biosciences (San Jose, CA, USA). After staining, cells were washed, resuspended, and analyzed by flow cytometry (33). At least 20,000 lymphocytes were identified by size (FSC) and granularity (SSC) and acquired using a FACS Canto II flow cytometer (BD Biosciences). All data were analyzed by the Flowjo 7.2.5 software (Tree Star Inc., Ashland, OR, USA).

### Statistical Analyses

All variables were tested for normality of distribution by Shapiro–Wilk test. Considering the median value (38.5) of the CSSA score among all patients with CUD included in this study, addicted individuals were divided in low withdrawal (L-W) and high withdrawal (H-W) severity groups. Thus, analysis were performed considering three groups: controls, L-W CUD, and H-W CUD. For categorical variables, differences between groups were compared using chi-square (X^2^) test. General linear models or Kruskal–Wallis were used to analyze differences between cocaine addicted groups and healthy non-addicted individuals. *Post-hoc* analysis were performed to compare differences between groups. Pearson and Spearman tests were used to correlation analysis. Statistical analysis were performed using the Statistical Package for Social Sciences, SPSS Statistics V.20 software (SPSS Inc., Chicago, IL, USA). The significance level was set at α = 0.05 (two tailed).

## Results

### Sociodemographic and Clinical Characteristics

Demographic and clinical data are depicted in Table 1. The majority of participants with CUD reported recent crack use (93%, *n* = 50), but 42% (*n* = 23) reported recent snorted cocaine use and 38% (*n* = 21) had used both smoked and snorted cocaine prior to treatment enrollment (last month). No participants reported injected cocaine use. In addition, although cocaine was reported as the primary abused substance and all individuals of the addicted group had a diagnosis of CUD, 98% (*n* = 52) of them were tobacco smokers, 70% (*n* = 37) cannabis abusers, and 36% (*n* = 19) reported chronic alcohol abuse. In the healthy control group, no lifetime history of substance abuse (alcohol, cannabis, and cocaine) was reported, excepted for tobacco (26%, *n* = 5).

**Table 1:** Demographic and clinical data.

	**Control group**	**Cocaine use disorder groups**		**Statistics**	***P*-value**
		**Low withdrawal**	**High withdrawal**		
Age (years)	30.50 (8.75)	33.00 (8.5)	34.00 (9.0)	3.692	0.158^a^
Income (US$)	701 (423)	383 (595)	370 (333)	3.420	0.181^a^
**Ethnicity**					
White	7 (39)	12 (48)	13 (52)	0.736	0.692^c^
Non-white	11 (61)	13 (52)	12 (48)		
BMI	27.37 (1.8)	23.23 (1.3)	23.23 (1.2)	29.274	<0.001^a^
CSSA	–	26.00 (13.5)	55.00 (15.5)	36.812	<0.001^b^
**Recent substance use**					
Cocaine	–	15.36 (8.0)	14.32 (9.9)	0.165	0.687^b^
Cannabis	–	5.00 (8.8)	6.68 (10.9)	0.333	0.567^b^
Alcohol	–	5.43 (8.3)	9.3 (10.9)	1.667	0.204^b^
Tobacco	2.30 (8.3)	20.58 (8.0)	19.68 (9.33)	21.868	<0.001^a^

*BMI, body mass index; CSSA, Cocaine Selective Severity Assessment. Recent substance use regarding 30 days prior to treatment enrollment. Data represented in median and interquartile range, or number of participants and percentage*.

a *Kruskal–Wallis*.

b *Mann–Whitney*.

c *Chi-square*.

*Controls—Non-addicted control group (n = 19); CUD addicted group—low withdrawal (n = 25) and high withdrawal (n = 25)*.

While no significant group differences were detected regarding age, income, and ethnicity, both CUD groups had significantly lower mean BMI compared with the healthy non-addicted control group. We therefore explored the relationships between BMI and immune markers and we did not find any significant associations (all *p* > 0.05, assessed by spearman's correlation).

### Cytokines

**Figure 1:**
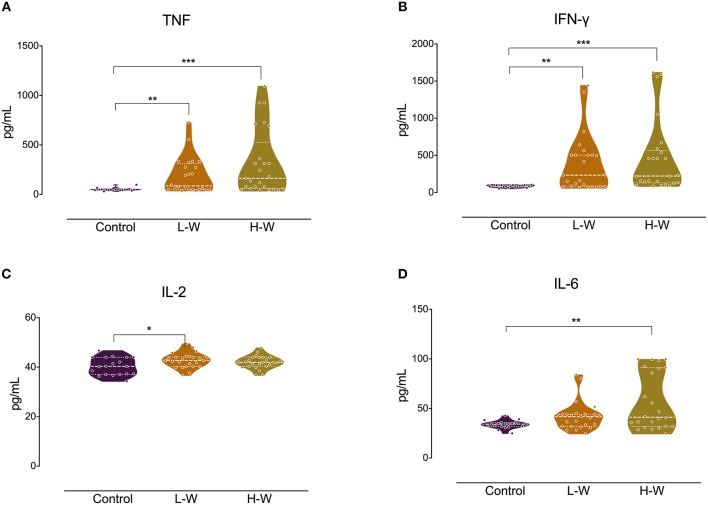
Th-1 cytokines after *in vitro* stimulation in women with CUD and healthy controls. PBMCs were seeded in 96-well plate and stimulated with phytohemagglutinin (1%) for 72 h. Cell supernatants collected from women with CUD had higher levels of TNF **(A)** and IFN-γ **(B)** than controls. Only L-W group cells shown increased release of IL-2 in comparison to controls **(C)**. Only H-W group cells showed higher levels of IL-6 compared to controls **(D)**. Kruskal–Wallis test was performed for TNF, IFN-γ, and IL-6 statistical analyses; data are shown as median with interquartile range. ANOVA followed by Bonferroni was used to IL-2 statistical analysis. Data are shown as mean ± SD. Statistical significant group differences are indicated ^*^p < 0.05, ^**^p < 0.01, and ^***^p < 0.001.

We investigated the production of Th1/Th2/Th17 cytokines *in vitro*. Analysis of Th1-related cytokines revealed that both CUD groups had higher levels of TNF-α (*H* = 17.94; *p* < 0.001; Figure 1A) and IFN-γ (*H* = 21.22; *p* < 0.001; Figure 1B) compared with controls, but no statistical significance was observed in pairwise comparisons between CUD groups (*p* > 0.05). A significant group difference was found for IL-2 levels (*F* = 3.59; *p* = 0.033; Figure 1C), with higher levels in the L-W group relative to controls (*p* = 0.030). No differences were found between CUD groups (*p* > 0.05). In contrast, only the H-W group had higher levels of IL-6 (*H* = 8.68; *p* < 0.013; Figure 1D) than controls, and L-W CUD group and controls had similar levels (*p* > 0.05).

**Figure 2:**
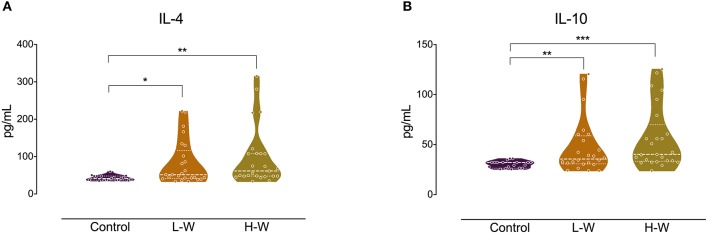
Th-1 cytokines after *in vitro* stimulation in women with CUD and healthy controls. PBMCs were seeded in 96-well plate and stimulated with phytohemagglutinin (1%) for 72 h. Cell supernatants collected from women with CUD showed higher levels of IL-4 **(A)** and IL-10 **(B)** than controls. Kruskal–Wallis test was carried out to compare statistical differences. Data are shown as median with interquartile range. Statistical significant group differences are indicated ^*^p < 0.05, ^**^p < 0.01, and ^***^p < 0.001.

**Figure 3:**
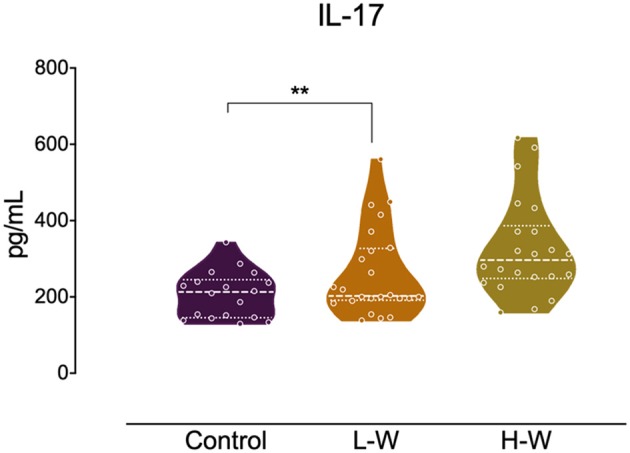
Th-17 related cytokine after *in vitro* stimulation in women with CUD and healthy controls. PBMCs were seeded in 96-well plate and stimulated with phytohemagglutinin (1%) for 72 h. Cell supernatants collected from L-W group had higher levels of IL-17 than controls. Kruskal–Wallis test was carried out to compare statistical differences. Data are shown as median with interquartile range. Statistical significant group differences are indicated. ^**^p < 0.01.

Analysis of Th2 cytokines revealed that both CUD groups had higher levels of IL-4 (*H* = 14.26; *p* < 0.001; Figure 2A) and IL-10 (*H* = 17.01; *p* < 0.001; Figure 2B) when compared with controls, but no statistical difference was observed between L-W and H-W groups (*p* > 0.05). In Th17-related cytokine, only L-W group exhibited higher levels of IL-17 compared to controls (*H* = 11.86; *p* < 0.002; Figure 3).

### Major Lymphocyte Subsets

**Figure 4:**
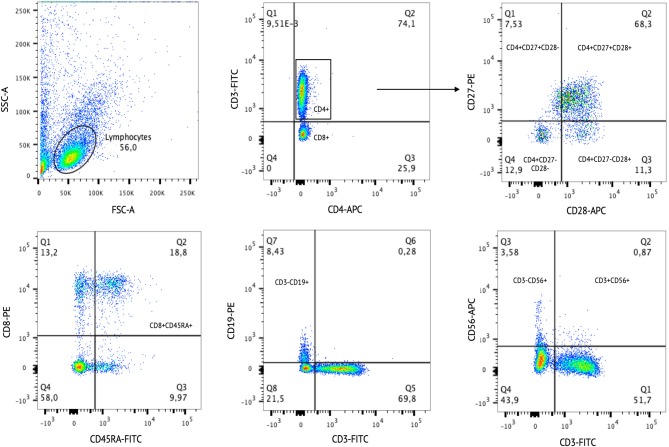
Example of cell gating strategy. The Side Scatter parameter (SSC-A) is correlated with cell granularity. The Forward Scatter parameter (FSC-A) is correlated with cell size. The SSC-A and FSC-A are used to select single cells out of the debris. After the cell populations were selected according to specific staining.

We investigated a comprehensive panel of major lymphocyte subpopulations, including T, B, and NK cells (Table 2). Figure 4 represents an example for gating strategy. Regarding T cells, both CUD groups had a lower percentage of naïve T helper lymphocytes (CD3^+^CD4^+^CD45RA^+^) compared with controls. However, the percentage of total helper T lymphocytes (CD3^+^CD4^+^) was significantly higher in the H-W group than in L-W addicted group and controls. Also, the H-W group had a lower percentage of naïve cytotoxic T lymphocytes (CD3^+^CD8^+^CD45RA^+^) compared with non-addicted controls.

**Table 2:** Immunophenotyping of lymphocyte subsets.

**Markers**	**Cell type**	**Control group**	**Cocaine use disorder groups**		**Statistics**	***P*-value**
			**Low withdrawal**	**High withdrawal**		
CD3^+^CD4^+^	Th	41.62 (2.1)^#^	41.97 (2.0)^#^	49.56 (2.3)^*^	4.070	**0.025**^a^
CD3^+^CD4^+^CD45RA^+^	Th naïve	19.74 (2.1)^#^	12.75 (1.9)^*^	12.20 (2.2)^*^	4.061	**0.025**^a^
CD3^+^CD4^+^CD45RO^+^	Th memory	38.40 (2.4)	42.70 (12.6)	41.90 (19.9)	1.668	0.434^b^
CD4^+^CD27^+^CD28^+^	Early-differentiated Th cell	73.34 (3.3)^#^	71.31 (3.2)^#^	59.57 (3.4)^*^	4.807	**0.014**^a^
CD4^+^CD27^−^CD28^+^	Intermediate-differentiated Th cell	1.59 (2.6)^#^	4.14 (9.9)	6.90 (5.7)^*^	6.123	**0.047**^b^
CD4^+^CD27^−^CD28^−^	Late-differentiated Th cell	3.78 (8.9)	10.50 (8.8)	6.79 (14.5)	3.047	0.218^b^
CD3^+^CD8^+^	Tc	19.70 (2.0)	17.52 (1.9)	15.09 (2.1)	1.245	0.299^a^
CD3^+^CD8^+^CD45RA^+^	Tc naïve	15.50 (12.5)^#^	14.60 (6.9)	10.00 (8.5)^*^	9.435	**0.009**^b^
CD3^+^CD8^+^CD45RO^+^	Tc memory	7.28 (1.1)	9.70 (1.0)	8.14 (1.1)	1.317	0.280^a^
CD8^+^CD27^+^CD28^+^	Early-differentiated Tc cell	44.33 (4.2)	45.31 (4.4)	48.55 (4.6)	0.244	0.785^a^
CD8^+^CD27^−^CD28^+^	Intermediate-differentiated Tc cell	12.39 (1.4)	11.18 (1.6)	11.79 (1.6)	0.159	0.853^a^
CD8^+^CD27^−^CD28^−^	Late-differentiated Tc cell	38.48 (4.9)	28.52 (5.1)	25.35 (5.3)	1.852	0.172^a^
CD4/CD8	Ratio	2.66 (0.4)	2.59 (0.3)	3.5 (0.4)	2.069	0.141^a^
CD3^+^CD25^+^CD69^+^	Activated T cell	0.75 (1.1)	1.25 (1.3)	0.81 (1.2)	0.938	0.626^b^
CD3^−^CD19^+^	B	9.77 (1.6)^#^	11.86 (1.5)	15.86 (1.7)^*^	3.460	**0.042**^a^
CD3^−^CD25^+^CD69^+^	Activated B cell	0.45 (0.8)	0.16 (0.4)	0.21 (0.6)	2.878	0.237^b^
CD3^−^CD56^+^	NK	5.49 (0.6)	4.01 (0.6)	4.31 (0.7)	1.467	0.243^a^
CD3^+^CD56^+^	NK T	1.07 (1.7)	0.92 (0.5)	0.78 (1.1)	0.116	0.944^b^

a *General linear model—Mean (Standard Error)*.

b *Kruskal–Wallis—Median (Interquartile Range)*.

*, #x00023;: *Indicate differences between groups (Tukey post-hoc test or Kruskal–Wallis post-hoc test). Th, helper T cell; Tc, cytotoxic T cell; NK, Natural Killer cell. Data represents the percentage of cells. Non-addicted control group (n = 14); CUD addicted group—low withdrawal (n = 15) and high withdrawal (n = 12)*.

*Bold values are related to significant associations, p <0.05*.

Different stages of T-cell differentiation were determined based on the cell-surface expression of the costimulatory molecules CD27 and CD28: naïve T cells or early-differentiated (CD27^+^CD28^+^), intermediate-differentiated (CD27^−^CD28^+^), and late-differentiated or senescent cells (CD27^−^CD28^−^). The H-W CUD group had a lower percentage of early-differentiated helper T cells (CD4^+^CD27^+^CD28^+^) compared with non-addicted controls, but no statistical significance was observed in the L-W group. In contrast, the percentage of intermediate-differentiated helper T cells (CD4^+^CD27^−^CD28^+^) was higher in the H-W group than in L-W addicted group and in controls.

Regarding B cells, only the H-W CUD group had a higher percentage of B cells (CD3^−^CD19^+^) compared to non-addicted controls, given that no statistical significance was observed between the L-W and control groups. No significant differences were found between groups for the remaining subsets.

Exploratory correlations showed that there is a positive association between CD3^+^CD4^+^ T cells and the amount of alcohol use in the last 30 days (*r* = 0.44, *p* = 0.027), but not with other variables impacted by cocaine use. Therefore, we included this variable as covariate in the CD3^+^CD4^+^ T cells between group comparisons. Indeed, we lost the effect when alcohol consume is covariate [*F*_(3, 41)_ = 2.27, *p* = 0.117]. We also found a positive association between CD4^+^CD27^−^CD28^+^ T cells and the amount of marijuana use in the last 30 days (*r* = 0.54, *p* = 0.005), but not with other variables impacted by cocaine use. Therefore, we included this variable as covariate in the CD4^+^CD27^−^CD28^+^ T cells between group comparisons. Again, we lost the significance when marijuana consume is covariate [*F*_(3, 39)_ = 1.66, *p* = 0.204]. No impact of tobacco in our immune variables was found.

Next, the expression of cell-surface markers, by the analysis of the mean fluorescence intensity (MFI), was compared between groups (Table 3). It was found that both CUD groups had lower expression of CD45RA in CD4 T lymphocytes as compared with controls.

**Table 3:** Mean fluorescence intensity (MFI) of activated and regulatory markers.

**Markers**	**Control group**	**Cocaine use disorder groups**		**Statistics**	***P*-value**
		**Low withdrawal**	**High withdrawal**		
**CD3**^**−**^					
CD19	683.50 (324.2)	507.00 (511.0)	607.00 (483.0)	0.533	0.766^b^
CD56	1562.00 (1218.7)	1660.00 (1042.0)	1346.00 (1021.0)	1.592	0.451^b^
**CD3**^**+**^					
CD25	441.50 (303.5)	249.00 (216.0)	305.00 (123.5)	1.148	0.563^b^
CD69	392.50 (319.5)	294.00 (332.0)	418.50 (334.2)	0.929	0.628^b^
**CD4**^**+**^					
CD45RA	5488.50 (4044.0)^#^	4256.00 (3875.0)^*^	4225.50 (4508.2)	7.869	**0.020**^**b**^
CD45RO	1557.00 (1242.5)	1616.00 (846.0)	1741.50 (719.7)	0.108	0.948^b^
CD27	1515.00 (1147.5)	1531.50 (790.7)	1450.00 (999.0)	0.207	0.902^b^
CD28	2464.50 (1833.0)	1791.00 (1392.5)	1749.00 (1340.0)	3.438	0.179^b^
**CD8**^**+**^					
CD45RA	4466.46 (542.9)	3579.47 (505.4)	2939.58 (565.0)	1.928	0.160^a^
CD45RO	1824.00 (906.0)	1836.00 (501.0)	1898.00 (769.0)	0.179	0.914^b^
CD27	913.00 (1172.7)	1215.00 (660.0)	955.00 (1034.0)	0.318	0.853^b^
CD28	701.50 (1661.7)	856.00 (714.0)	1107.50 (898.2)	2.315	0.314^b^

a *General linear model—Mean (Standard Error)*.

b *Kruskal–Wallis—Median (Interquartile Range)*.

*,# *Indicate differences between groups (Kruskal–Wallis post-hoc test). Non-addicted control group (n = 14); CUD group—low withdrawal (n = 15) and high withdrawal (n = 12)*.

*Bold values are related to significant associations, p <0.05*.

## Discussion

The evaluation of inflammatory biomarkers linked to addiction-related outcomes may help establishing a prognosis and identify subgroups of patients who will struggle more severely with cocaine withdrawal symptoms (26, 29). In our study, we investigated possible immunological differences between healthy women and women with CUD, taking into account different severity (low or high) of withdrawal symptoms. For that, we investigate for the first time a large immunophenotyping panel, which reveal some differences in lymphocytes population (T and B cells) between CUD and healthy controls. Moreover, we elucidated that both proinflammatory and anti-inflammatory PHA-induced cytokines are exacerbated in CUD (mainly in high withdrawal) group compared to healthy controls.

Overall, addicted individuals showed increased levels of Th1, Th2, and Th17 cytokines in comparison to control group. However, higher levels of IL-2 and IL-17 were observed only in the L-W group, while increased levels of IL-6 were detected only in the H-W group. Regarding lymphocyte subsets, some notable differences were detected between the three groups analyzed. The H-W group presented increase percentage of CD3^+^CD4^+^ T cells and B cells, and lower percentage of naïve CD3^+^CD8^+^ T cells. Both groups of individuals with CUD had lower a percentage of naïve CD3^+^CD4^+^ T cells compared to controls, while specific differences were observed in relation to T-cell differentiation.

Inflammatory processes are intimately correlated to the production of cytokines, which are released in response to infections, drug use, or neuroinflammation, and can cross the blood-brain barrier, reaching the peripheral blood (34, 35). Cytokines can also influence the synthesis, release, and reuptake of neurotransmitters, such as dopamine, leading to effects in reward and withdrawal-related regions of the brain, and act as mediators between the immune system and the CNS (34, 36). Some cytokines, including IFN-γ and IL-4, may influence the regulation of specific neuronal circuits related to memory and social behavior (37, 38).

Furthermore, cocaine use increases dopamine levels in the brain and periphery, and through dopamine transporter binding on peripheral lymphocytes, may modulate immunological mechanisms (39). Also, experimental studies demonstrated that lesions in the nigrostriatal dopaminergic system can cause increase in the number of T and B cells in the periphery (40). T cells have an important role in CNS injury, they can acquire Th1 and Th17 phenotypes, release cytokines such as IFN-γ and IL-17, thus leading to tissue damage. On the other hand, T cells may acquire a Th2 phenotype, producing IL-4 and promoting neuroprotection (41). These relationships offer an interesting opportunity to understand the complex network related to drug addiction and biological effects.

In the present study, only PBMCs from individuals with L-W symptoms showed increased IL-2 cytokine production compared to control, after *in vitro* stimulation with PHA 1% for 72 h. There is evidence that cocaine may have an influence on cytokine production, and this is closely related to the dose used. *In vitro* experiments have shown that high doses of cocaine (100–200 ng/mL) can inhibit the IL-2 cytokine production induced by Concanavalin A in mouse spleen cells (42). Also, mitogen-stimulated splenocytes from mice that received acute or chronic cocaine exposure, exhibited decreased production of IL-2, IFN-gamma, and IL-4 (43). Concerning PHA stimulated PBMCs, was observed that individuals with higher concentration of cocaine in hair also had high levels of IL-2 production by PBMCs (44). It allow us to postulate that perhaps individuals with L-W symptoms had more circulating cocaine metabolites than H-W group.

Furthermore, regarding IL-17 cytokine levels, here only PBMCs from L-W group showed increased production compared to control. Otherwise, when IL-17 was evaluated directly in plasma samples, it was verified that cocaine addicted subjects presented lower levels of this cytokine compared with no addicted control group. However, when cocaine addicted subjects were divided in two sub-groups according to cocaine symptoms severity, there was no differences in IL-17 plasma levels between mild/moderate and severe withdrawal scores (45). This discrepancy in the results can be attributed to the methodological differences between the present study and the study conducted by Maza-Quiroga et al. (45).

Recently, Moreira et al. (16), found increased levels of IL-6 and decreased levels of IL-10 in serum samples of cocaine users (males and females) compared to controls, demonstrating an inflammatory effect of cocaine. In this sense, cocaine use can lead to behavior and neuronal responses through central immune signaling perturbation (46). Corroborating these findings, we observed higher production of IL-6 in the H-W group, suggesting that IL-6 could be an immune biomarker of worse clinical outcome among individuals with CUD, particularly related to severe withdrawal symptoms during early drug abstinence. *In vitro* and *in vivo* models showed that IL-6 can regulate synaptic transmission and synaptic plasticity, participating in mechanisms involved in cognition, memory, and learning (47). In addition, IL-6 can be produced by several cell types, but B cells are the main lymphoid source. This molecule act regulating the proliferation and differentiation of B cells (48), supporting our findings that higher IL-6 levels could be correlated with increase expression of B cells in the H-W group. Also, heavy alcoholics had lower numbers of peripheral B cells than moderate and light drinkers (49) and patients with alcoholic liver disease showed a significant depletion in B cell population compared to controls (50).

To the best of our knowledge, few studies have evaluated the effects of substance use in specific lymphocyte populations, and even fewer considering cocaine abuse. Cocaine modifies the functioning of immune cells, including NK and T cells, neutrophils, and macrophages (18, 51). In addicted individuals, overall results concerning number and percentage of such cells are still inconsistent. For instance, elevated percentage of T CD4^+^ cells were observed in individuals with cocaine use (52, 53), corroborating our findings; however, other studies did not find such association (15). In addition, alcohol exposure was related to a decline in total T CD4^+^ cells (54).

Decreased numbers of naïve T cells were observed in chronic and heavy alcohol consumption, which can be converted into memory cells (55), in accordance with our findings in women with CUD. Other studies have showed that cocaine exposure elevated the percentage of naïve T CD8^+^ and total T CD8^+^ cells and decreased the percentage of T CD8^+^ memory cells subpopulation (15, 53, 56). Alcohol exposure can also lead to overexpression of activated T CD8^+^ cells (54). Regarding NK cells, data remains inconsistent. We did not observe any alteration in NK cell population, as Ruiz et al. (57) in contrast, other studies with cocaine users even detected an increase (15, 56) as well as a decrease in NK cells (50, 52).

Our study revealed that most changes in lymphocyte subsets occur in individuals with more withdrawal symptoms. Of note, we observed that in the H-W group there is a higher percentage of total T CD4^+^ cells and T CD4^+^ lymphocytes in the initial stage of differentiation, but a lower percentage of naïve T CD4^+^ cells. The CD4/CD8 ratio is considered an indicator of immune system effectiveness, and it has been used to diagnosis and monitor viral infections, such as HIV and hepatitis (58, 59). In animal models, cocaine administration leads to lymphopenia, with a significant decline in T, B, and NK cells, causing a disproportional number of lymphocytes subsets and resulting in elevated CD4/CD8 ratio in peripheral blood (60). Here, although we observed alterations in lymphocytes subpopulations, the CD4/CD8 ratio did not differ between CUD individuals and controls.

This study has some limitations. Considering the influence of sex in addiction, it is necessary to be careful in extrapolating findings to males. For instance, women present higher vulnerability, worse clinical outcomes and faster scalation to drug dependence (61, 62). Also, our sample size is moderate, especially in the control group. However, healthy control individuals included in this study were carefully recruited, with restricted criteria including absence of any psychiatry disorder, no previous history of substance abuse and no medication use for at least 30 days. In addition, our clinical sample was restricted to drug addicts, since participants were comprehensively assessed and fulfilled all diagnostic criteria for CUD. Finally, some studies have evaluated cytokine *in vitro* concentration using PHA and/or LPS to stimulate PBMC's. However, due to the small cell amount in our samples and the strong correlation of cytokine concentration among serum and PHA-stimulated supernatants, we used only PHA.

Considering the polydrug use profile in our sample, we explore possible associations between drug use (i.e., alcohol, marijuana, and tobacco) during 30 days before treatment admission and immune cell types. The positive correlation found with alcohol and marijuana use, can indicate the overall contribution of these drugs to trigger an inflammatory response during the withdrawal period. Disruption in immune system homeostasis was already showed in alcohol use dependents, which exhibited an increase of activated CD8^+^ T cells compared to healthy subjects (54). Moreover, acute alcohol withdrawal was associated to increase the TNF-α levels which can evoke an inflammatory response (63).

Despite marijuana use, THC can impact human cell-mediated immunity and host defenses (64). A previous study suggested that cannabis exerts a cytotoxic on immune cells, where cannabis consumption causes impairments in cell functions and cell proliferation, resulting in decreased numbers of CD8^+^ T and NK cells compared to controls (65). However, marijuana can exerts a dual effect on the immune system, acting as anti-inflammatory considering the acute exposure or in long-lasting exposure triggers a proinflammatory outcome on brain cytokines (66). Nonetheless, the molecular mechanisms underlying polydrug use impact on the immune system remain poorly understood.

In conclusion, our results suggest that lymphocytes from women with CUD are responsive to PHA stimulus and release higher amounts of both pro-inflammatory and anti-inflammatory cytokines compared to controls. Interestingly enough, IL-6 was found higher only in H-W group, suggesting that cytokine is closely related to CUD severity symptoms. Also, cocaine use leads to a perturbation in immune T and B cells phenotypes, mainly in individuals with high withdrawal symptoms.

## Data Availability Statement

The raw data supporting the conclusions of this manuscript will be made available by the authors, without undue reservation, to any qualified researcher. Requests to access the datasets should be directed to RG-O ( rodrigo.grassi@pucrs.br).

## Ethics Statement

The study protocol was approved by both scientific and ethics committees 39868314.0.0000.5336 of PUCRS (Porto Alegre, Brazil) and written informed consent was obtained from all participants.

## Author Contributions

The paper was written by AZ, JS, and TV and revised into its final format by all co-authors. Sample collection and PBMCs isolation were conducted by AZ, TB, and AB. CBA performance and analysis was conducted by AZ, JS, and CP. Immunophenotyping and cytometric analysis were performed by AZ, JS, TB, and MB. Statistical analysis was performed by AZ, JS, TV, and RG-O. Clinical assessment was conducted by BS-V. All authors read and approved the final manuscript.

### Conflict of Interest

The authors declare that the research was conducted in the absence of any commercial or financial relationships that could be construed as a potential conflict of interest.

## References

[1] Abdalla RR Madruga CS Ribeiro M Pinsky I Caetano R Laranjeira R. Prevalence of cocaine use in Brazil: data from the II Brazilian National Alcohol and Drugs Survey (BNADS). Addict Behav. (2014) 39:297–301. 10.1016/j.addbeh.2013.10.019 24455783

[2] Hall FS Sora I Drgonova J Li X-F Goeb M Uhl GR. Molecular mechanisms underlying the rewarding effects of cocaine. Ann NY Acad Sci. (2004) 1025:47–56. 10.1196/annals.1316.006 15542699

[3] Hyman SE Malenka RC Nestler EJ. Neural mechanisms of addiction: the role of reward-related learning and memory. Annu Rev Neurosci. (2006) 29:565–98. 10.1146/annurev.neuro.29.051605.113009 16776597

[4] Fox HC D'Sa C Kimmerling A Siedlarz KM Tuit KL Stowe R. Immune system inflammation in cocaine dependent individuals: implications for medications development. Hum Psychopharmacol. (2012) 27:156–66. 10.1002/hup.1251 22389080 PMC3674778

[5] Levandowski ML Viola TW Tractenberg SG Teixeira AL Brietzke E Bauer ME. Adipokines during early abstinence of crack cocaine in dependent women reporting childhood maltreatment. Psychiatry Res. (2013) 210:536–40. 10.1016/j.psychres.2013.07.007 23896356

[6] Zaparte A Viola T Grassi-Oliveira R da Silva Morrone M Moreira J Bauer M. Early abstinence of crack-cocaine is effective to attenuate oxidative stress and to improve antioxidant defences. Psychopharmacology. (2014) 232:1405–13. 10.1007/s00213-014-3779-8 25338778

[7] Sordi AO Pechansky F Henrique F Kessler P Kapczinski F Kessler FH. Oxidative stress and BDNF as possible markers for the severity of crack cocaine use in early withdrawal. Psychopharmacology. (2014) 231:4031–9. 10.1007/s00213-014-3542-1 24676990

[8] Zubaran C Foresti K Thorell MR Franceschini PR Rossi M Roberto P. Anxiety symptoms in crack cocaine and inhalant users admitted to a psychiatric hospital in Southern Brazil. Rev Assoc Med Bras. (2013) 59:360–7. 10.1016/j.ramb.2013.01.008 23850024

[9] Koppel BS Samkoff L Daras M. Relation of cocaine use to seizures and epilepsy. Epilepsia. (1996) 37:875–8. 10.1111/j.1528-1157.1996.tb00041.x 8814101

[10] Gordon RJ Lowy FD. Bacterial infections in drug users. N Engl J Med. (2005) 353:1945–54. 10.1056/NEJMra042823 16267325

[11] Wilson T DeHovitz JA. STDs, HIV, and crack cocaine: a review. AIDS Patient Care STDS. (1997) 11:62–6. 10.1089/apc.1997.11.62 11361764

[12] DeBeck K Kerr T Li K Fischer B Buxton J Montaner J. Smoking of crack cocaine as a risk factor for HIV infection among people who use injection drugs. Can Med Assoc J. (2009) 181:585–9. 10.1503/cmaj.082054 19841052 PMC2764753

[13] Levite M. Dopamine and T cells: dopamine receptors and potent effects on T cells, dopamine production in T cells, and abnormalities in the dopaminergic system in T cells in autoimmune, neurological and psychiatric diseases. Acta Physiol. (2016) 216:42–89. 10.1111/apha.12476 25728499

[14] Basu S Dasgupta PS. Dopamine, a neurotransmitter, influences the immune system. J Neuroimmunol. (2000) 102:113–24. 10.1016/S0165-5728(99)00176-9 10636479

[15] Ruiz P Cleary T Nassiri M Steele B. Human T lymphocyte subpopulation and NK cell alterations in persons exposed to cocaine. Clin Immunol Immunopathol. (1994) 70:245–50. 10.1006/clin.1994.1036 7508835

[16] Moreira FP Medeiros JRC Lhullier AC Souza LD de M Jansen K Portela LV. Cocaine abuse and effects in the serum levels of cytokines IL-6 and IL-10. Drug Alcohol Depend. (2016) 158:181–5. 10.1016/j.drugalcdep.2015.11.024 26679059

[17] Narvaez JCM Magalhaes PV Fries GR Colpo GD Czepielewski LS Vianna P. Peripheral toxicity in crack cocaine use disorders. Neurosci Lett. (2013) 544:80–4. 10.1016/j.neulet.2013.03.045 23597759

[18] Irwin MR Olmos L Wang M Valladares EM Motivala SJ Fong T. Cocaine dependence and acute cocaine induce decreases of monocyte proinflammatory cytokine expression across the diurnal period: autonomic mechanisms. J Pharmacol Exp Ther. (2006) 320:507–15. 10.1124/jpet.106.112797 17068203

[19] Levandowski ML Viola TW Prado CH Wieck A Bauer ME Brietzke E. Distinct behavioral and immunoendocrine parameters during crack cocaine abstinence in women reporting childhood abuse and neglect. Drug Alcohol Depend. (2016) 167:140–8. 10.1016/j.drugalcdep.2016.08.010 27530287

[20] Araos P Pedraz M Serrano A Lucena M Barrios V García-Marchena N. Plasma profile of pro-inflammatory cytokines and chemokines in cocaine users under outpatient treatment: influence of cocaine symptom severity and psychiatric co-morbidity. Addict Biol. (2015) 20:756–72. 10.1111/adb.12156 24854157

[21] Almeida PP de Araujo Filho GM Malta SM Laranjeira RR Marques ACRP Bressan RA. Attention and memory deficits in crack-cocaine users persist over four weeks of abstinence. J Subst Abuse Treat. (2017) 81:73–8. 10.1016/j.jsat.2017.08.002 28847458

[22] Johnson JE O'Leary CC Striley CW Abdallah AB Bradford S Cottler LB. Effects of major depression on crack use and arrests among women in drug court. Addiction. (2011) 106:1279–86. 10.1111/j.1360-0443.2011.03389.x 21306595 PMC3711247

[23] Helmus TC Downey KK Wang LM Rhodes GL Schuster CR. The relationship between self-reported cocaine withdrawal symptoms and history of depression. Addict Behav. (2001) 26:461–7. 10.1016/S0306-4603(00)00105-2 11436938

[24] Montgomery SL Bowers WJ. Tumor necrosis factor-alpha and the roles it plays in homeostatic and degenerative processes within the central nervous system. J Neuroimmune Pharmacol. (2012) 7:42–59. 10.1007/s11481-011-9287-2 21728035

[25] Salim S Chugh G Asghar M. Inflammation in anxiety. Adv Protein Chem Struct Biol. (2012) 88:1–25. 10.1016/B978-0-12-398314-5.00001-5 22814704

[26] Sukoff Rizzo SJ Neal SJ Hughes ZA Beyna M Rosenzweig-Lipson S Moss SJ. Evidence for sustained elevation of IL-6 in the CNS as a key contributor of depressive-like phenotypes. Transl Psychiatry. (2012) 2:e199. 10.1038/tp.2012.120 23212583 PMC3565187

[27] Kampman KM Volpicelli JR McGinnis DE Alterman AI Weinrieb RM D'Angelo L. Reliability and validity of the cocaine selective severity assessment. Addict Behav. (1998) 23:449–61. 10.1016/S0306-4603(98)00011-2 9698974

[28] Dluzen DE McDermott JL. Sex differences in dopamine- and vesicular monoamine-transporter functions. Ann NY Acad Sci. (2008) 1139:140–50. 10.1196/annals.1432.010 18991858

[29] Elman I Karlsgodt KH Gastfriend DR. Gender differences in cocaine craving among non-treatment-seeking individuals with cocaine dependence. Am J Drug Alcohol Abuse. (2001) 27:193–202. 10.1081/ADA-100103705 11417935

[30] First MB Williams JBW Karg RS Spitzer RL Structured Clinical Interview for DSM-5 Disorders, Clinician Version (SCID-5-CV). Arlington, VA: American Psychiatric Association (2016).

[31] Kessler F Cacciola J Alterman A Faller S Souza-Formigoni ML Cruz MS. Psychometric properties of the sixth version of the Addiction Severity Index (ASI-6) in Brazil. Rev Bras Psiquiatr. (2012) 34:24–33. 10.1590/S1516-44462012000100006 22392385

[32] Taylor AL Llewelyn MJ. Superantigen-induced proliferation of human CD4^+^CD25- T cells is followed by a switch to a functional regulatory phenotype. J Immunol. (2010) 185:6591–8. 10.4049/jimmunol.1002416 21048104

[33] Trintinaglia L Bandinelli LP Grassi-Oliveira R Petersen LE Anzolin M Correa BL. Features of immunosenescence in women newly diagnosed with breast cancer. Front Immunol. (2018) 9:1651. 10.3389/fimmu.2018.01651 30061900 PMC6055359

[34] Cisneros IE Erdenizmenli M Cunningham KA Paessler S Dineley KT. Cocaine evokes a profile of oxidative stress and impacts innate antiviral response pathways in astrocytes. Neuropharmacology. (2018) 135:431–43. 10.1016/j.neuropharm.2018.03.019 29578037 PMC5975185

[35] Ghosh A Birngruber T Sattler W Kroath T Ratzer M Sinner F. Assessment of blood-brain barrier function and the neuroinflammatory response in the rat brain by using cerebral open flow microperfusion (cOFM). PLoS ONE. (2014) 9:e98143. 10.1371/journal.pone.0098143 24852285 PMC4031165

[36] Kampman KM Pettinati HM Volpicelli JR Oslin DM Lipkin C Sparkman T. Cocaine dependence severity predicts outcome in outpatient detoxification from cocaine and alcohol. Am J Addict. (2004) 13:74–82. 10.1080/10550490490265389 14766440

[37] Derecki NC Cardani AN Yang CH Quinnies KM Crihfield A Lynch KR. Regulation of learning and memory by meningeal immunity: a key role for IL-4. J Exp Med. (2010) 207:1067–80. 10.1084/jem.20091419 20439540 PMC2867291

[38] Filiano AJ Xu Y Tustison NJ Marsh RL Baker W Smirnov I. Unexpected role of interferon-γ in regulating neuronal connectivity and social behaviour. Nature. (2016) 535:425–9. 10.1038/nature18626 27409813 PMC4961620

[39] Sarkar C Basu B Chakroborty D Dasgupta PS Basu S. The immunoregulatory role of dopamine: an update. Brain Behav Immun. (2010) 24:525–8. 10.1016/j.bbi.2009.10.015 19896530 PMC2856781

[40] Engler H Doenlen R Riether C Engler A Niemi M-B Besedovsky HO. Time-dependent alterations of peripheral immune parameters after nigrostriatal dopamine depletion in a rat model of Parkinson's disease. Brain Behav Immun. (2009) 23:518–26. 10.1016/j.bbi.2009.01.018 19486644

[41] Filiano AJ Gadani SP Kipnis J. How and why do T cells and their derived cytokines affect the injured and healthy brain? Nat Rev Neurosci. (2017) 18:375–84. 10.1038/nrn.2017.39 28446786 PMC5823005

[42] Wang Y Huang DS Watson RR. *In vivo* and *in vitro* cocaine modulation on production of cytokines in C57BL/6 mice. Life Sci. (1994) 54:401–11. 10.1016/0024-3205(94)00698-9 8295487

[43] Di Francesco P Marini S Pica F Favalli C Tubaro E Garaci E. *In vivo* cocaine administration influences lymphokine production and humoral immune response. Immunol Res. (1992) 11:74–9. 10.1007/BF02918610 1602184

[44] Guan-Jie C Pillai R Erickson JR Martinez F Estrada AL Watson RR Cocaine immunotoxicity: abnormal cytokine production in hispanic drug users. Toxicol Lett. (1991) 59:81–8. 10.1016/0378-4274(91)90058-E 1721732

[45] Maza-Quiroga R García-Marchena N Romero-Sanchiz P Barrios V Pedraz M Serrano A. Evaluation of plasma cytokines in patients with cocaine use disorders in abstinence identifies transforming growth factor alpha (TGFα) as a potential biomarker of consumption and dual diagnosis. PeerJ. (2017) 5:e3926. 10.7717/peerj.3926 29038767 PMC5641428

[46] Coller JK Hutchinson MR. Implications of central immune signaling caused by drugs of abuse: mechanisms, mediators and new therapeutic approaches for prediction and treatment of drug dependence. Pharmacol Ther. (2012) 134:219–45. 10.1016/j.pharmthera.2012.01.008 22316499

[47] Gruol DL. IL-6 regulation of synaptic function in the CNS. Neuropharmacology. (2014) 96:42–54. 10.1016/j.neuropharm.2014.10.023 25445486 PMC4446251

[48] Matsushita T Regulatory and effector B cells: Friends or foes? J Dermatol Sci. (2019) 93:2–7. 10.1016/j.jdermsci.2018.11.008 30514664

[49] Sacanella E Estruch R Gayà A Fernández-Solà J Antúnez E Urbano-Márquez A. Activated lymphocytes (CD25^+^ CD69^+^ cells) and decreased CD19^+^ cells in well-nourished chronic alcoholics without ethanol-related diseases. Alcohol Clin Exp Res. (1998) 22:897–901. 10.1111/j.1530-0277.1998.tb03886.x 9660319

[50] Matos LC Batista P Monteiro N Ribeiro J Cipriano MA Henriques P. Lymphocyte subsets in alcoholic liver disease. World J Hepatol. (2013) 5:46. 10.4254/wjh.v5.i2.46 23646229 PMC3642723

[51] Stefanidou M Loutsidou AC Chasapis CT Spiliopoulou CA. Immunotoxicity of cocaine and crack. Curr Drug Abuse Rev. (2011) 4:95–7. 10.2174/1874473711104020095 21696343

[52] Ersche KD Döffinger R Inflammation and infection in human cocaine addiction. Curr Opin Behav Sci. (2017) 13:203–9. 10.1016/j.cobeha.2016.12.007

[53] Gan X Zhang L Newton T Chang SL Ling W Kermani V. Cocaine infusion increases interferon-γ and decreases interleukin-10 in cocaine-dependent subjects. Clin Immunol Immunopathol. (1998) 89:181–90. 10.1006/clin.1998.4607 9787120

[54] Zuluaga P Sanvisens A Martínez-Cáceres E Teniente A Tor J Muga R. Over-expression of CD8^+^ T-cell activation is associated with decreased CD4^+^ cells in patients seeking treatment of Alcohol Use Disorder. Drug Alcohol Depend. (2017) 180:7–13. 10.1016/j.drugalcdep.2017.07.023 28850904

[55] Cho BK Rao VP Ge Q Eisen HN Chen J. Homeostasis-stimulated proliferation drives naive T cells to differentiate directly into memory T cells. J Exp Med. (2000) 192:549–56. 10.1084/jem.192.4.549 10952724 PMC2193235

[56] Van Dyke C Stesin A Jones R Chuntharapai A Seaman W. Cocaine increases natural killer cell activity. J Clin Invest. (1986) 77:1387–90. 10.1172/JCI112445 2937807 PMC424504

[57] Ruiz P Berho M Steele BW Hao L. Peripheral human T lymphocyte maintenance of immune functional capacity and phenotypic characteristics followingin vivococaine exposure. Clin Immunol Immunopathol. (1998) 88:271–6. 10.1006/clin.1998.4579 9743614

[58] Ray K Gupta SM Bala M Muralidhar S Kumar J. CD4/CD8 lymphocyte counts in healthy, HIV-positive individuals & AIDS patients. Indian J Med Res. (2006) 124:319–30. 17085836

[59] Dimitropoulou D Karakantza M Tsamandas AC Mouzaki A Theodorou G Gogos CA. T-lymphocyte subsets in peripheral blood and liver tissue of patients with chronic hepatitis B and C. In Vivo. (2011) 25:833–40. 21753143

[60] Jankowski MM Ignatowska-Jankowska B Glac W Swiergiel AH. Cocaine administration increases CD4/CD8 lymphocyte ratio in peripheral blood despite lymphopenia and elevated corticosterone. Int Immunopharmacol. (2010) 10:1229–34. 10.1016/j.intimp.2010.07.003 20637837

[61] Becker JB Perry AN Westenbroek C. Sex differences in the neural mechanisms mediating addiction: a new synthesis and hypothesis. Biol Sex Differ. (2012) 3:14. 10.1186/2042-6410-3-14 22676718 PMC3724495

[62] Becker JB McClellan ML Reed BG. Sex differences, gender and addiction. J Neurosci Res. (2017) 95:136–47. 10.1002/jnr.23963 27870394 PMC5120656

[63] Freeman K Brureau A Vadigepalli R Staehle MM Brureau MM Gonye GE. Temporal changes in innate immune signals in a rat model of alcohol withdrawal in emotional and cardiorespiratory homeostatic nuclei. J Neuroinflammation. (2012) 9:598. 10.1186/1742-2094-9-97 22626265 PMC3411448

[64] Oláh A Szekanecz Z Bíró T. Targeting cannabinoid signaling in the immune system: “High”-ly exciting questions, possibilities, and challenges. Front Immunol. (2017) 8:1487. 10.3389/fimmu.2017.01487 29176975 PMC5686045

[65] Tanasescu R Constantinescu CS. Cannabinoids and the immune system: an overview. Immunobiology. (2010) 215:588–97. 10.1016/j.imbio.2009.12.005 20153077

[66] Moretti S Castelli M Franchi S Raggi MA Mercolini L Protti M Δ^9^-Tetrahydrocannabinol-induced anti-inflammatory responses in adolescent mice switch to proinflammatory in adulthood. J Leukoc Biol. (2014) 96:523–34. 10.1189/jlb.3HI0713-406RR 24744434

